# Development and validation of a circulating microRNA panel for the early detection of breast cancer

**DOI:** 10.1038/s41416-021-01593-6

**Published:** 2022-01-10

**Authors:** Ruiyang Zou, Sau Yeen Loke, Yew Chung Tang, Heng-Phon Too, Lihan Zhou, Ann S. G. Lee, Mikael Hartman

**Affiliations:** 1 Department of Research and Development, MiRXES Lab, Singapore, Singapore; 2 Cellular and Molecular Research, Humphrey Oei Institute of Cancer Research, National Cancer Centre, Singapore, Singapore; 3 Department of Biochemistry, Yong Loo Lin School of Medicine, National University of Singapore, Singapore, Singapore; 4 NUS Center for Cancer Research, Yong Loo Lin School of Medicine, National University of Singapore, Singapore, Singapore; 5 SingHealth Duke-NUS Oncology Academic Clinical Programme, Duke-NUS Medical School, Singapore, Singapore; 6 Department of Physiology, Yong Loo Lin School of Medicine, National University of Singapore, Singapore, Singapore; 7 Department of Surgery, Yong Loo Lin School of Medicine, National University of Singapore and National University Health System, Singapore, Singapore; 8 Saw Swee Hock School of Public Health, National University of Singapore and National University Health System, Singapore, Singapore

**Keywords:** Tumour biomarkers, Breast cancer, Cancer screening, Diagnostic markers

## Abstract

**Background:**

Mammography is widely used for breast cancer screening but suffers from a high false-positive rate. Here, we perform the largest comprehensive, multi-center study to date involving diverse ethnic groups, for the identification of circulating miRNAs for breast cancer screening.

**Methods:**

This study had a discovery phase (*n* = 289) and two validation phases (*n* = 374 and *n* = 379). Quantitative PCR profiling of 324 miRNAs was performed on serum samples from breast cancer (all stages) and healthy subjects to identify miRNA biomarkers. Two-fold cross-validation was used for building and optimising breast cancer-associated miRNA panels. An optimal panel was validated in cohorts with Caucasian and Asian samples. Diagnostic ability was evaluated using area under the curve (AUC) analysis.

**Results:**

The study identified and validated 30 miRNAs dysregulated in breast cancer. An optimised eight-miRNA panel showed consistent performance in all cohorts and was successfully validated with AUC, accuracy, sensitivity, and specificity of 0.915, 82.3%, 72.2% and 91.5%, respectively. The prediction model detected breast cancer in both Caucasian and Asian populations with AUCs ranging from 0.880 to 0.973, including pre-malignant lesions (stage 0; AUC of 0.831) and early-stage (stages I–II) cancers (AUC of 0.916).

**Conclusions:**

Our panel can potentially be used for breast cancer screening, in conjunction with mammography.

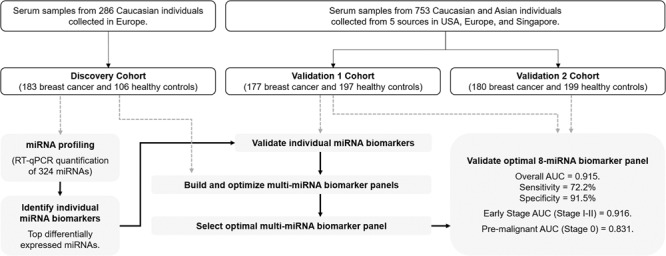

## Background

Mammography has been widely-used as a screening tool for breast cancer despite its high false-positive rate, and its lack of sensitivity in detecting cancer in dense breasts [1]. A high rate of false positivity of 11–12% has been detected among women in the United States who have undergone mammographic screening [2, 3]. Minimally invasive methods such as miRNA-based liquid biopsies can potentially overcome these disadvantages and improve overall detection accuracy [4, 5]. MiRNAs are deemed suitable as biomarkers because of altered miRNA expression profiles in cancer that reflect disease development, as well as the stability and the accessibility of circulating miRNAs in a myriad of body fluids including blood, urine and saliva [6, 7].

MiRNAs are evolutionary conserved, single-stranded non-coding RNAs of 19–25 nucleotides that primarily function in mediating the degradation or translational repression of mRNA targets [7]. Under normal physiological conditions, miRNAs are key components of feedback mechanisms for a wide range of biological pathways such as cell proliferation, differentiation and apoptosis [8]. Conversely, dysregulated miRNAs have been implicated in carcinogenesis including supporting tumour growth by inhibiting growth suppression, sustaining proliferative signalling and resisting cell death, activating invasion and metastasis, and promoting angiogenesis [6]. It is now known that miRNAs regulate oncogenesis through their tumour suppressor or oncogenic activities, with increasing evidence of aberrant miRNA expression in a variety of malignancies [9].

In an effort to improve breast cancer detection, numerous promising blood-derived miRNA biomarkers with superior discriminative ability as compared to mammography, have been reported in recent years [1, 10, 11]. The miRNAs miR-145, miR-21 and miR-221 are among the more frequently reported candidates and demonstrate potential for the early detection of breast cancer [12–15]. However, despite these findings, these breast cancer-associated miRNA biomarker studies are still at the discovery phase, with none successfully transitioned to clinical trials. This could be due to various shortcomings. For example, the majority of previously published circulating miRNA biomarker studies for breast cancer were conducted in smaller sample sizes comprising of a single ethnic group, and with one or no validation phase. Several studies on blood-based circulating miRNA signatures were generated based on one discovery cohort, with the sample size of less than 200 study subjects without subsequent validation cohorts [11–14, 16]. Other studies had only one validation phase each [17–20]. A prospective validation using 116 women [21] was carried out as a follow-up to the study by Kodahl et al. [18]. However, in the prospective study, the nine-miRNA signature (miR-15a, miR-18a, miR-107, miR-133a, miR-139-5p, miR-143, miR-145, miR-365 and miR-425) failed to differentiate between the non-cancer controls and the ER+ or the ER− subtypes (AUC of 0.580 and 0.610, respectively) [21], possibly due to insufficient validation of the miRNA signature in the previous retrospective study.

Here, we have carried out the largest comprehensive, multi-center study to date involving diverse ethnic groups comprising of Caucasians and Asians, for the identification of circulating miRNAs associated with breast cancer. Expression profiles of serum miRNAs were elucidated from several independent cohorts of breast cancer cases and non-cancer controls following an analysis workflow that accounted for possible pre-analytic confounding factors. The performance of potential circulating miRNAs was established in three phases, with a discovery cohort and two independent validation cohorts. Hence, we are the first to report a miRNA-based breast cancer prediction model with high discriminative ability in classifying breast cancer patients from non-cancer individuals, and with the capability of identifying breast cancer in both Caucasian and Asian populations.

## Methods

### Study design and cohorts

This multi-centre case-control study was carried out in three phases: one discovery phase and two validation phases. The three patient cohorts used (Discovery Cohort, Validation 1 Cohort, and Validation 2 Cohort, respectively) comprised samples from six different sources (Table 1). The Discovery cohort comprised European Caucasian samples obtained from the Asterand biobank (designated as Source 1) while the Validation 1 and 2 cohorts were a mix of Caucasian and Asian samples from five different sources in USA, Ukraine, Russia, and Singapore (designated as Sources 2–5) (Table 1). The Discovery and Validation cohorts included female subjects diagnosed with stage 1 to stage 3 breast cancer of all subtypes (Supplementary Table S1). The inclusion criteria for cases was women diagnosed with breast cancer, and the inclusion criteria for controls was no history of cancer in healthy female individuals. A blinded approach was not done as our study design included two validation phases. Written informed consent was obtained from all participants and the research was approved by all relevant Institutional Review Boards (IRBs). Samples from the Asterand and Tissue Solution biobanks were ethically collected under IRB-approved protocols and fully consented.

**Table 1:** Patient cohorts used in study.

Cohort:			Discovery		Validation 1		Validation 2	
*Non-cancer (NC)/Cancer (C):*			NC	C	NC	C	NC	C
			106	183	197	177	199	180
*Source*		*Ethnicity*						
1	Asterand biobank (Europe)	Caucasian	106 (100%)	183 (100%)	–	–	–	–
2	Asterand biobank (USA)	Caucasian	–	–	39 (19%)	39 (22%)	39 (19%)	40 (22%)
3	Tissue Solutions biobank (Ukraine)	Caucasian	–	–	33 (17%)	23 (13%)	34 (17%)	24 (13%)
4	Tissue Solutions biobank (Russia)	Caucasian	–	–	47 (24%)	48 (27%)	47 (24%)	48 (27%)
5	National University Hospital	Asian (Singapore)	–	–	35 (18%)	38 (21%)	35 (18%)	38 (21%)
6	National Cancer Centre Singapore	Asian (Singapore)	–	–	43 (22%)	29 (16%)	44 (22%)	30 (17%)
		*Age (years)*						
		Mean	53.7	52.4	53.0	56.6	55.5	55.4
		Median	53	51	52	57	56	55
		Range	42–65	30–85	29–82	31–77	26–83	28–87
		*Sex*						
		Male	0	0	0	0	0	0
		Female	106	183	197	177	199	180
		*Cancer Stage*						
		0	–	0 (0%)	–	16 (9%)	–	23 (13%)
		I	–	77 (42%)	–	51 (29%)	–	45 (25%)
		II	–	78 (43%)	–	61 (34%)	–	58 (32%)
		III	–	28 (15%)	–	5 (3%)	–	8 (4%)
		IV	–	0 (0%)	–	3 (2%)	–	3 (2%)
		Unknown	–	0 (0%)	–	41 (23%)	–	43 (24%)

### Blood collection and serum processing

Peripheral blood samples (20 ml) were drawn from subjects using venipuncture and collected in serum tubes. Blood samples were clotted for 30–60 min and were centrifuged at 1300 rcf at room temperature for 20 min. Sera were then aliquoted for immediate storage at −80 °C.

### RNA Isolation

Total RNA was extracted from 200 µl of each serum sample using the miRNeasy Serum/Plasma Kit (Qiagen, Hilden, Germany). This was done according to the manufacturer’s recommendations, except for the following modifications: (a) a set of three proprietary spike-in controls (MiRXES, Singapore) was added, representing high, medium, and low levels of RNA, into the sample lysis buffer (QIAzol Lysis Reagent, Qiagen) prior to sample RNA isolation. The spike-in controls are 20-nucleotide RNAs with unique sequences (distinct from any of the 2588 annotated mature human miRNAs in miRBase version 21.0, RRID:SCR_003152) and are used to monitor RNA isolation efficiency and normalise for technical variations during RNA isolation; (b) bacteriophage MS2 RNA was added into sample lysis buffer (1 µg per ml of QiaZol) to improve RNA isolation yield; (c) the samples were centrifuged at 18,000 × *g* for 15 min at room temperature after mixing with chloroform; and finally, (d) the RNA was eluted in 25 µl of RNase-free water.

### RT-qPCR Detection of miRNA expression

For biomarker discovery, a highly-controlled RT-qPCR workflow was used to quantify the expression of 324 miRNAs in each serum sample (Supplementary Table S2). Serum RNA was reverse transcribed using miRNA-specific reverse transcription (RT) primers according to manufacturer’s instructions (ID3EAL Customized Individual miRNA RT Primer, MiRXES) on a Veriti™ Thermal Cycler (Applied Biosystems, Foster City, CA, USA). Multiplexed RT reactions were carried out using specific RT primers for 324 miRNAs. This proprietary list of 324 circulating miRNAs was selected based on experimental analysis of more than 1000 high confidence human miRNAs from several hundred serum and plasma specimens. These 324 miRNAs are therefore those which have been detected with high confidence in human serum and plasma samples. The RT primers were divided into 10 multi-plex primer pools (50–60-plex per pool) to minimise non-specific cross-overs and primer-primer interactions. For each RNA sample, 10 multiplex RT reactions were performed, each with 2 µl of isolated RNA. Synthetic templates for standard curves of each miRNA (6-log serial dilution of 10 million to 100 copies) and a non-template control (nuclease-free water spiked with MS2) were reverse transcribed concurrently with the isolated sample RNA. Synthetic miRNA standard curves were used to absolutely quantify sample miRNA expression copy numbers. To measure 324 miRNAs using quantitative PCR (qPCR), all cDNAs, including those from synthetic miRNA standards, were pre-amplified using a 14-cycle PCR reaction with Augmentation Primer Pools (MiRXES) on the Veriti™ Thermal Cycler. Single-plex qPCR was then performed on the amplified cDNA samples using a miRNA-specific qPCR assay (MiRXES) and ID3EAL miRNA qPCR Master Mix according to manufacturer’s instructions (MiRXES). The qPCR reactions with technical duplicates were carried out on the ViiA™ qPCR system (384-well configuration, Applied Biosystems). Raw threshold cycle (Ct) values were calculated using the ViiA™ 7 RUO software with automatic baseline setting and a threshold of 0.5. RT-qPCR efficiency and potential cDNA amplification bias were assessed by analysing the Ct values of the synthetic miRNA standards. The absolute expression of each miRNA (number of copies present) in the serum sample was calculated by intrapolation of sample Ct values with synthetic miRNA standard curves and correcting for variations in RT-qPCR efficiency. For biomarker validation, miRNA expression was quantified using the same workflow described above, adjusted for the number of miRNAs to be quantified.

### Biomarker discovery

The absolute quantities of 324 candidate miRNAs in the serum of both breast cancer cases and non-cancer controls were determined. The geNORM (geNORM, RRID:SCR_006763) [22] and NormFinder (NormFinder, RRID:SCR_003387) [23] software were used to identify endogenous reference miRNAs that had stable expression across all samples and could be used to normalise for varying sample RNA inputs for RT-qPCR. Three miRNAs with stable expression were identified and used to normalise the expression levels of miRNAs across samples: miR-128-3p, miR-652-3p, and miR-106b-3p (Supplementary Fig. S1). The normalised miRNA expression values were used to compare the expression levels of individual miRNAs between breast cancer cases and non-cancer controls. Unsupervised hierarchical clustering was carried out based on Euclidean distance of normalised miRNA expression levels in two dimensions (samples and miRNA expression). The top miRNAs with *p* < 0.01 and magnitude of log2 fold change >0.5 were selected for validation using the Validation 1 cohort. Statistical significance of differences in miRNA expression was determined using Student’s *t*-test. All p-values were corrected for multiple hypotheses testing using false discovery rate (FDR) adjustment [24, 25]. Those miRNAs which were differentially expressed in both the Discovery and Validation 1 cohorts were considered validated. A relaxed cut-off of *p* < 0.05 with magnitude of log2 fold change >0.5 was used to identify validated miRNAs for biomarker panel building and optimisation.

### Biomarker panel building and optimisation

A two-fold cross-validation procedure that incorporated the sequential forward floating search (SFFS) algorithm [26] and a logistic regression model was used for building and optimising miRNA biomarker panels to discriminate between breast cancer cases and non-cancer controls. Starting from an empty panel, SFFS progressively searches for the next best marker to add to the panel already chosen. It is a search strategy to arrive at an optimal panel without conducting an exhaustive search of all possible combinations. For each iteration of the cross-validation procedure, SFFS will arrive at one optimal panel (one optimal two-miRNA panel, one optimal three-miRNA panel, and so on). The SFFS was used to select miRNA biomarkers for inclusion in each biomarker panel built. In each iteration of the two-fold cross validation procedure, the samples included in the combined Discovery and Validation 1 cohorts (comprising a total of 663 samples from six sources) were randomly partitioned into two equal groups: Group A and Group B. The proportion of subjects from each of the six sources were partitioned equally in both Group A and B. During each iteration of cross-validation, Group A was first used as the training set for building a breast cancer prediction model while Group B was used as the test set. The group assignments as training and testing sets were then swapped. For every multi-miRNA biomarker panel optimised in each iteration, a logistic regression prediction model was built and the diagnostic ability of each panel was evaluated using the area under the curve of the receiver operating characteristics (AUC) analysis. The cross-validation procedure was carried out 200 times. Thus, 200 two-miRNA panels, 200 three-miRNA panels, and so on, were optimised and tested. The diagnostic power (AUC) of each optimised multi-miRNA panel for classifying breast cancer and non-cancer patient samples was then calculated and compared with other panels optimised in each iteration. Using a logistic regression model incorporating multi-miRNA biomarker panel expression measurements, a prediction algorithm score could be calculated for each sample, with higher scores indicating increased risk of cancer. A prediction algorithm score cut-off was then used to predict breast cancer.

## Results

### Discovery and validation of significant differentially regulated miRNAs

**Fig. 1:**
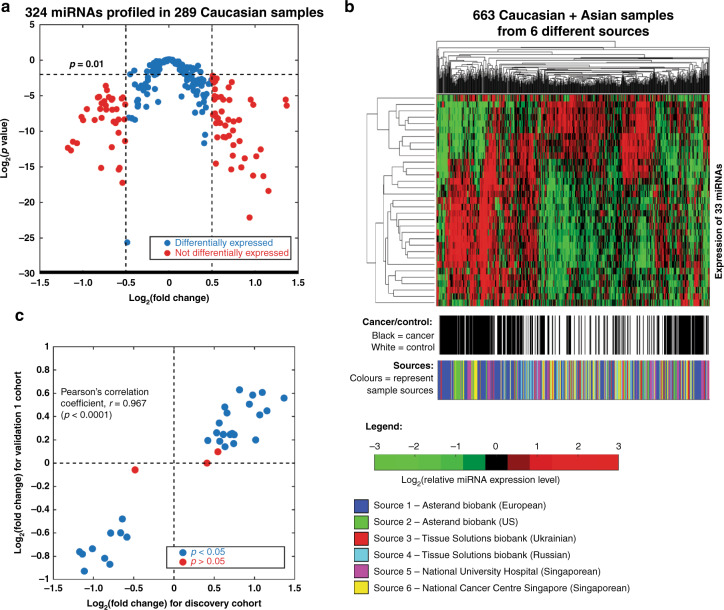
Biomarker discovery. **a** Volcano plot for 324 miRNAs profiled in 183 breast cancer patients as compared to 106 healthy individuals in the discovery cohort. Eighty-six miRNAs with *p*-values of less than 0.01 and magnitudes of log2 (fold change) of more than 0.5 are highlighted in red. **b** Heat-map of 663 cancer and non-cancer samples clustered using the expression of 33 selected miRNA biomarkers identified in the Discovery cohort. The expression levels (copy/ml) of miRNAs are presented in log2 scale and standardised to zero mean. The colour scale represents the concentrations of miRNA. Hierarchical clustering was carried out for both dimensions (miRNAs: *Y*-axis, samples: *X*-axis) based on the Euclidean distance. **c** Correlation of log2 (fold change) for 33 selected miRNA biomarker candidates in the Discovery cohort and Validation 1 cohort. The miRNA biomarker candidates with *p*-values larger than 0.05 in the validation 1 cohort are highlighted in red.

The expression levels of 324 miRNAs, which have been previously detected with high confidence in human serum, were quantified in the Discovery Cohort of 289 Caucasian samples (183 breast cancer samples and 106 non-cancer controls). All samples in this cohort were obtained from a single source as shown in Table 1. MiRNAs that were significantly differentially expressed between breast cancer cases and non-cancer controls were identified by *p*-value of unpaired Student’s *t*-test and fold change in expression. A total of 86 miRNAs that were differentially expressed between cancer cases and non-cancer controls with log2 (fold change) more than 0.5 or less than −0.5 and *p-*value < 0.01 were identified (Fig. 1a).

The ability of these 86 differentially expressed miRNAs to differentiate between breast cancer cases and non-cancer controls was also assessed using AUC analysis. Out of these 86 differentially expressed miRNAs, 33 miRNAs had AUC > 0.5 and were selected for validation in a mixed Caucasian-Asian cohort (Validation 1). This cohort comprised of 374 samples (177 breast cancer cases and 197 non-cancer controls) from five different sources (three Caucasian and two Asian populations) as shown in Table 1.

Unsupervised hierarchical clustering based on the differential expressions of the 33 top-ranked miRNA biomarker candidates was carried out on the combined Discovery and Validation 1 cohort (663 samples comprising 360 breast cancer cases and 303 non-cancer controls). The cancer samples and the non-cancer samples were partially separated after clustering based on differential expression of these 33 miRNAs (Fig. 1b). Additionally, samples from the same sources were not clustered together based on their expression of these 33 miRNAs (Fig. 1b).

The log2 (fold change) calculated for these 33 miRNA biomarker candidates in the Discovery cohort and Validation 1 cohort were compared and had a Pearson’s correlation coefficient, *r* = 0.967 (*p* < 0.0001). Out of the 33 biomarker candidates identified from the Discovery cohort, 30 miRNAs were differentially expressed in breast cancer cases compared to non-cancer controls (*p* < 0.05 by unpaired *t*-test) in the Validation 1 cohort (Fig. 1c). Three miRNA biomarker candidates that showed non-significant differential expressions (*p* > 0.05) in Validation 1 cohort were excluded from the subsequent analysis, while the remaining 30 miRNA biomarker candidates were used for the biomarker panel optimisation phase.

### Optimisation of miRNA biomarker panels

**Fig. 2:**
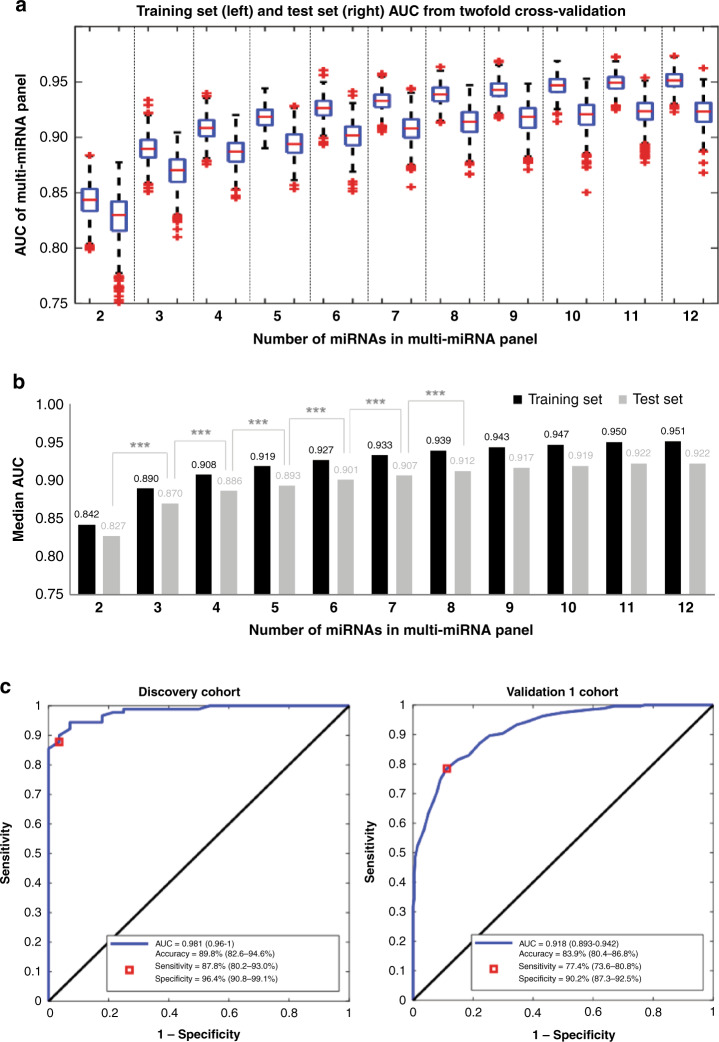
Optimisation of multi-miRNA biomarker panels. **a** Boxplots of AUC of multi-miRNA biomarker panels (with 2–12 miRNAs), in both the training and test sets, calculated from 200 iterations of the two-fold cross-validation process. The boxplot presents the 25th, 50th, and 75th percentiles in panel AUC. **b** Median AUC for the training and test sets from 200 iterations of the two-fold cross-validation process for multi-miRNA panels with 2–12 miRNAs. ***p < 0.001 (Student’s *t*-test). **c** ROC curves for breast cancer prediction performance of the optimal eight-miRNA biomarker panel in the Discovery and Validation 1 cohorts. The point with the maximum classification accuracy is shown as the red box. The sensitivity and specificity values at the maximum accuracy point are also shown. The 95% CI for these values is shown in the brackets.

To identify an optimal panel with good performance while balancing the number of miRNAs included for the practicality of clinical testing, multi-miRNA panels were assessed. The best-performing multi-miRNA panel comprising between two to twelve miRNAs were formed from the 30 validated miRNA biomarker candidates using a two-fold cross-validation procedure that incorporated a feature selection algorithm (SFSS). AUC of miRNA panel performance in the training and test group was calculated for 200 iterations of cross-validation with multi-miRNA panels comprising two to twelve miRNAs (Fig. 2a). The median AUC for breast cancer prediction from 200 iterations of training and testing was calculated for each set of cross-validation experiments comprising two to twelve miRNAs (Fig. 2b). The median AUC increased significantly (*p* < 0.001) with increasing number of miRNAs in the biomarker panels that consisted of two to eight miRNAs, until it reached a plateau after the inclusion of eight miRNAs on the panel (Fig. 2b); hence indicating that eight miRNAs is the optimal number of biomarkers to be included on the panel. The addition of more miRNAs did not lead to a statistically significant increase in AUC. The optimal eight-miRNA biomarker panel with the highest AUCs of 0.981 and 0.918 in the Discovery and Validation 1 cohorts, respectively, was chosen for further validation (Fig. 2c). This optimal panel included miR-133a-3p, miR-497-5p, mir-24-3p, and miR-125b-5p, which were upregulated in breast cancer cases compared to controls, and miR-377-3p, miR-374c-5p, miR-324-5p and miR-19b-3p, which were downregulated in breast cancer cases as compared to controls (Supplementary Table S3). At the point of maximum classification accuracy, sensitivity and specificity were 87.8% (95% CI, 80.2–93.0%) and 96.4% (95% CI, 90.8–99.1%) in the Discovery Cohort, and 77.4% (95% CI, 73.6–80.8%) and 90.2% (95% CI, 87.3–92.5%) in the Validation 1 Cohort (Fig. 2c).

### Validation of optimal eight-miRNA biomarker panel signature

**Fig. 3:**
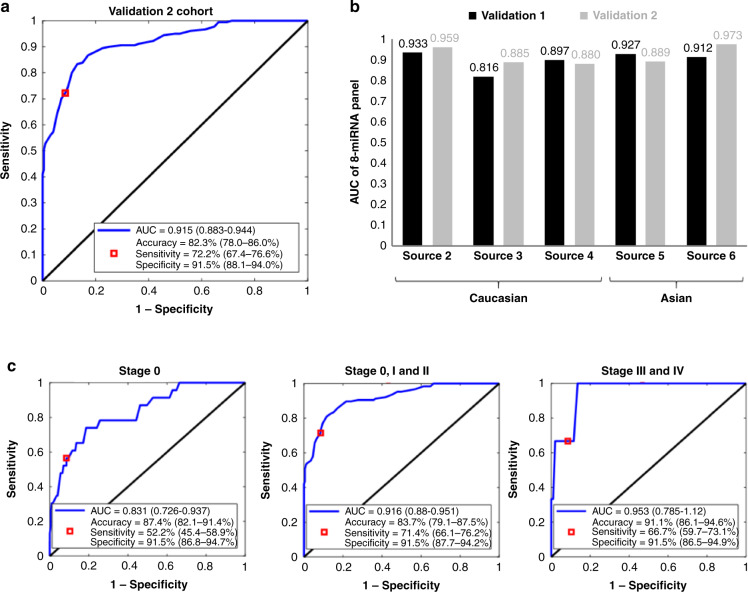
Validation of optimal eight-miRNA biomarker panel. **a** ROC curve for breast cancer prediction performance of the optimal eight-miRNA biomarker panel in the Validation 2 cohort. The point with the maximum classification accuracy is shown as the red box. The sensitivity and specificity values at the maximum accuracy point are also shown. The 95% CI for these values is shown in the brackets. **b** AUC of eight-miRNA biomarker panel in detecting breast cancer in the Validation 1 and Validation 2 cohorts separated by sample source. **c** ROC curves for performance of the optimal eight-miRNA biomarker panel in predicting early (stages 0, I and II) and late (stages III and IV) breast cancer in the Validation 2 cohort.

Validation of the optimised eight-miRNA biomarker panel signature was carried out in the Validation 2 cohort which comprised of 379 samples (180 breast cancer and 199 non-cancer samples). The AUC of the eight-miRNA biomarker panel in classifying breast cancer and non-cancer samples was 0.915 (95% CI, 0.883–0.944) (Fig. 3a). At the point of maximum classification accuracy, sensitivity was 72.2% (95% CI, 67.4–76.6%) with a specificity of 91.5% (95% CI, 88.1–94.0%). When the Validation 1 and 2 cohorts were separated by sample source, the eight-miRNA biomarker panel had AUC ranging from 0.816 to 0.973 (Fig. 3b). Performance was comparable between Caucasian and Asian sample sources (Fig. 3b) and for early stage (stages 0, I and II) and late stage (stages III and IV) breast cancers (Fig. 3c).

### Breast cancer prediction algorithm based on miRNA biomarker signature

**Fig. 4:**
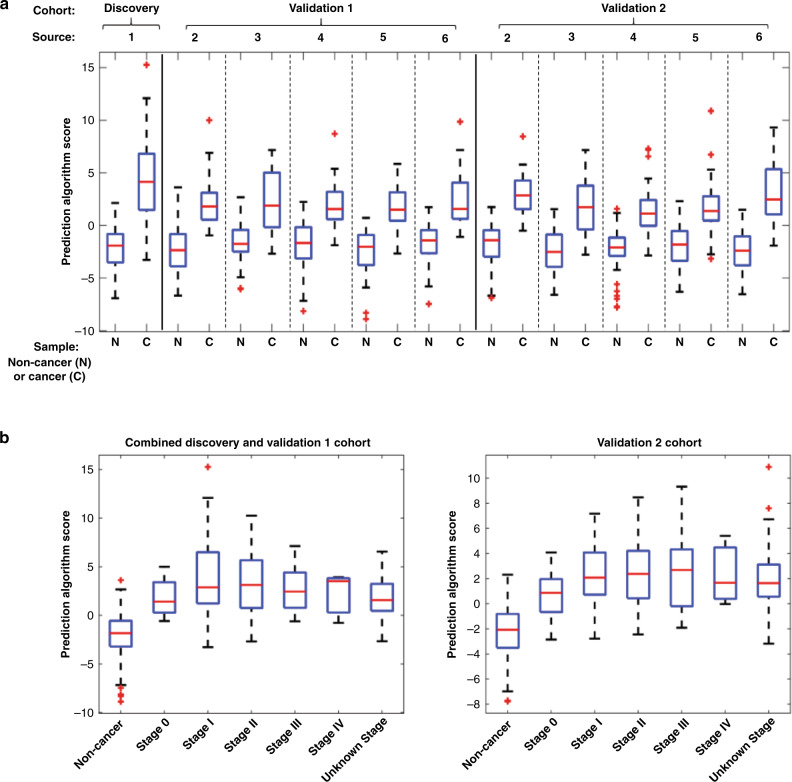
Prediction algorithm based on the expression of eight-miRNA biomarker panel. **a** Prediction algorithm scores of cancer and non-cancer samples calculated from the expression of eight-miRNA panel expression. **b** Prediction algorithm scores of non-cancer samples and cancer samples by tumour stage (0, I, II, III, IV, and unknown).

A prediction algorithm based on a logistic regression model that takes into account the expression levels of the eight miRNAs in the biomarker panel was developed to calculate a cancer risk score. The median cancer risks score calculated from the prediction algorithm were higher for cancer samples compared to non-cancer samples across all cohorts regardless of sample source (Fig. 4a). The panel effectively detects breast cancer of all stages, including early stage breast cancers (stages 0, I and II) (Fig. 3c), with cancer risk scores from breast cancer samples of all stages falling in the same range that is higher than that of non-cancer samples (Fig. 4b). The distribution of breast cancer samples by stage in each cohort is shown in Table 1.

## Discussion

We describe here an eight-miRNA biomarker signature that can differentiate between breast cancer patients and non-cancer individuals, which was derived from a multi-ethnic study comprising of a discovery phase followed by two validation phases. Our miRNA-based signature could perform well in distinguishing breast cancer cases from non-cancer controls as evidenced by a high AUC of 0.915 achieved in validation phase 2, which comprised of both Caucasians and Asians. These results imply that the performance of our model is robust, accurate and effective in classifying individuals diagnosed with breast cancer from those who are cancer-free. Therefore, to our knowledge, the present study is the first to date to describe the largest comprehensive multicenter study for the development of a circulating miRNA-based breast cancer prediction model that is applicable to both Caucasian and Asian populations.

The performance of the present miRNA-based prediction model for the initial discovery and two validation phases were consistent, as demonstrated by their respective AUCs of 0.981, 0.918 and 0.915. Likewise, when the validation phases 1 and 2 were analysed based on the sub-cohorts obtained from different sample sources, the range of AUCs generated in these sub-cohorts for both phases were comparable, ranging from 0.816 to 0.933 in phase 1 and from 0.880 to 0.973 in phase 2. These results highlight the high reproducibility and accuracy of our model in differentiating breast cancer cases from non-cancer controls for both Caucasians and Asians, suggesting its potential universal usability for various ethnicities. Our current findings are an important advancement for breast cancer miRNA biomarker research as our study design, analysis and results are unprecedented in the field.

Among the eight miRNAs in our signature, miR-133a-3p, miR-497-5p, mir-24-3p, and miR-125b-5p were found to be upregulated, whereas miR-377-3p, miR-374c-5p, miR-324-5p and miR-19b-3p were found to be downregulated in breast cancer cases as compared to controls. All of these miRNAs, except miR-133a-3p, have been reported to be implicated in the pathophysiology of breast cancer. For example, both miR-24-3p and miR-125b-5p have been identified as potential breast cancer biomarkers for the early detection [27, 28], prognosis [29, 30], or prediction of recurrence [31, 32]. Among the eight miRNAs discovered, there are discrepancies reported between the present and previous studies regarding the expression levels of miR-497-5p in breast cancer. Based on our current observation, miR-497-5p was upregulated in the serum samples of breast cancer patients whereas several studies have reported the decreased expression of miR-497-5p in breast cancer tissue samples and cell lines [33–36]. In a nude mouse xenograft tumour model, the inhibitory role of miR-497-5p in tumour growth and angiogenesis has been demonstrated [33] while low miR-497-5p expression was associated with poor prognosis of breast cancer patients [34]. For miR-377-3p, studies have shown that miR-377-3p was one of the miRNA transcripts that could predict tumour progesterone status with 100% accuracy [37] and the Linc00339/miR-377-3p/HOXC6 axis represented a novel pathway in the progression of triple-negative breast cancer [38]. MiR-374-5p has been shown to repress development of breast cancer through TATA-box binding protein associated factor 7 (TAF7)-mediated transcriptional regulation of DEP domain containing 1 (DEPDC1) [39]. The expression of miR-374-5p was downregulated in various breast cancer cell lines [39], similar to our observation in this study. A six-miRNA signature which included miR-324-5p has been discovered to be significantly associated with the reduced overall survival of triple-negative breast cancer [40]. MiR-19b-3p has also been shown to be downregulated in hormone receptor-positive/HER2-negative breast cancer [41]. With its high sensitivity and specificity in identifying breast cancer from healthy tissues and its the involvement in regulation of genes in oncogenic pathways, miR-19b-3p may serve as a diagnostic marker or therapeutic target for breast cancer [41].

Although our model could not outperform the five-miRNA signature (miR-1246, miR-1307-3p, miR-4634, miR-6861-5p and miR-6875-5p) model reported by the largest breast cancer miRNA biomarker study to date with an AUC of 0.971, it is noted that the latter was based primarily on microarray profiling, a method which is known to have poor specificity [42], and that only one miRNA, miR-1246, was validated by qRT-PCR using 26 serum samples [10]. For the performance comparison of the prediction model in a Singaporean Chinese cohort, our present model was able to achieve a better classification (AUC of 0.973) than the two-miRNA combinations generated from miR-1, miR-92a, miR-133a and miR-133b that were previously published (AUC of 0.900–0.910) [43]. In another study, a plasma signature of miR-145 and miR-451 was identified and demonstrated an outstanding ability in distinguishing breast cancer patients from healthy controls (AUC of 0.931) following a blind validation [15]. Although Ng et al. [15] conducted the study using multiple cohorts in different phases [17], the total sample size was smaller than the present study. In addition, a recent study reported a six-miRNA signature (miR-3124-5p, miR-1184, miR-4423-3p, miR-4529-3p, miR-7855-5p, and miR-4446-3p) with an AUC of 0.896 for discriminating high-risk women who eventually diagnosed with breast cancer from those who remained cancer-free [44]. However, a downside of this study was that the model was only tested in a single cohort of 48 samples [44].

Another observation from these studies is that there is a lack of strong overlap of miRNAs between studies which could possibly be attributed to differences between studies in sample type (whole blood, plasma or serum), timing of blood collection (before or after surgery), technology platform (microarray, RT-PCR or next-generation sequencing), study design and differences in data analysis. Hence, this suggests that for biomarker discovery research, having multiple validation cohorts is of utmost importance in order to verify the biomarker signature.

This study utilised qRT-PCR for miRNA profiling, since qRT-PCR is deemed as the gold standard for nucleic acid quantification due to the sensitivity and specificity of the method [42, 45]. Standard curves were also used to determine the absolute expression (copy number) of each miRNA target. In addition, since qRT-PCR is commonly utilised in various multigene prognostic assays including Onco*type* DX [46], Breast Cancer Index [47], and EndoPredict [48], this makes our miRNA-based breast cancer prediction model more readily translatable as a molecular diagnostic assay for clinical use.

Apart from miRNA biomarkers, there are other notable research efforts assessing alternative blood-based bioanalytes for breast cancer detection, such as the CancerSEEK study [49] and the Circulating Cell-Free Genome Atlas (CCGA) study [50]. CancerSEEK is a pan-cancer blood test intended for the identification of eight cancer types including breast cancer, by evaluating mutations in 16 genes from cell-free DNA (cfDNA) and the expression of eight protein biomarkers using multiplex PCR followed by next-generation sequencing and immunoassays respectively [49]. Similarly, the CCGA study which is an on-going prospective longitudinal cohort study that has enrolled approximately 15,000 study participants, also aims to develop a multi-cancer detection blood test by profiling cfDNA using sequencing-based methods [50]. Although these assays have been tested to detect different cancer types and stages, their performance for identifying breast cancer, especially in the early stages, is still under par. For the CancerSEEK test, the median detection sensitivity for breast cancer was 33% as compared to 98% for ovarian cancer, whereas the median detection sensitivity for stage I of all cancer types was only 43% as compared to 78% for stage III cancers [49]. Moreover, the tests developed by the CCGA study were poor in identifying various breast cancer molecular subtypes with sensitivities below 60% [51]. In contrast, our miRNA-based model showed superior discrimination performance, even for differentiating between healthy controls and those at pre-malignant stages (stage 0) with the AUC, accuracy, sensitivity and specificity of 0.831, 87.4%, 52.2% and 91.5%, respectively. In addition, the AUC and sensitivity increased to 0.916 and 71.4%, respectively for the detection of the pre-malignant stage and early-stage breast cancers (stages 0–II).

There are limitations or challenges in this current study. Firstly, while the present circulating miRNA-based prediction model performed well in identifying breast cancer cases, the specificity of the model for breast cancer as compared to other cancer types warrants further investigation [1]. Secondly, pre-analytical confounding factors might be present in the study given that these samples were sourced from different clinical centers and some pre-analytical factors such as sample processing procedure, storage duration, and transport condition could differ across the centers [1]. Hence, for future studies, a standardised protocol for sample collection, transport, processing, and storage should be used to minimise possible confounding factors that may lead to experimental biasness in the analysis of the predictive performance of the model. Thirdly, as the present study was conducted retrospectively, it is essential to further validate the miRNA-based prediction model in a prospective or blinded study and to benchmark the accuracy, sensitivity and specificity of our model against the current gold standard for breast cancer screening [1]. Such studies could be done on individuals who participate in routine annual or biennial mammogram screening, to ascertain its predictive value through comparison with mammography in a clinical setting. Future studies could also assess the performance of this panel in detecting breast cancers belonging to various subtypes, such as triple-negative breast cancers.

Together, this present study has generated a robust prediction model for breast cancer, applicable for Caucasian and Asian populations and patients of various cancer stages. The present miRNA-based prediction model can be potentially developed as an alternative modality for breast cancer screening, and may reduce the number of biopsies resulting from false-positive mammograms.

### Supplementary information

Supplementary Figure S1

Supplementary Table S1

Supplementary Table S2

Supplementary Table S3

## Data Availability

The datasets used and analysed in this study are available from the corresponding authors on reasonable request.
