# Correction: Development and validation of a circulating microRNA panel for the early detection of breast cancer

**DOI:** 10.1038/s41416-024-02800-w

**Published:** 2024-08-27

**Authors:** Ruiyang Zou, Sau Yeen Loke, Yew Chung Tang, Heng-Phon Too, Lihan Zhou, Ann S. G. Lee, Mikael Hartman

**Affiliations:** 1 Department of Research and Development, MiRXES Lab, Singapore, Singapore; 2 Cellular and Molecular Research, Humphrey Oei Institute of Cancer Research, National Cancer Centre, Singapore, Singapore; 3 Department of Biochemistry, Yong Loo Lin School of Medicine, National University of Singapore, Singapore, Singapore; 4 NUS Center for Cancer Research, Yong Loo Lin School of Medicine, National University of Singapore, Singapore, Singapore; 5 SingHealth Duke-NUS Oncology Academic Clinical Programme, Duke-NUS Medical School, Singapore, Singapore; 6 Department of Physiology, Yong Loo Lin School of Medicine, National University of Singapore, Singapore, Singapore; 7 Department of Surgery, Yong Loo Lin School of Medicine, National University of Singapore and National University Health System, Singapore, Singapore; 8 Saw Swee Hock School of Public Health, National University of Singapore and National University Health System, Singapore, Singapore

**Keywords:** Tumour biomarkers, Breast cancer, Cancer screening, Diagnostic markers

Correction to: *British Journal of Cancer* 10.1038/s41416-021-01593-6, published online 10 January 2022

The authors omitted to include the following Acknowledgements section in their article. It is being included here to provide the reader with the full list of contributions made to the study outlined in the article.

