# Supplementary figures and images for: Development and validation of a circulating microRNA panel for the early detection of breast cancer
Source: Br J Cancer. 2022 Jan 10;126(3):472–81. doi: 10.1038/s41416-021-01593-6 (PMC8810862; doi:10.1038/s41416-021-01593-6)

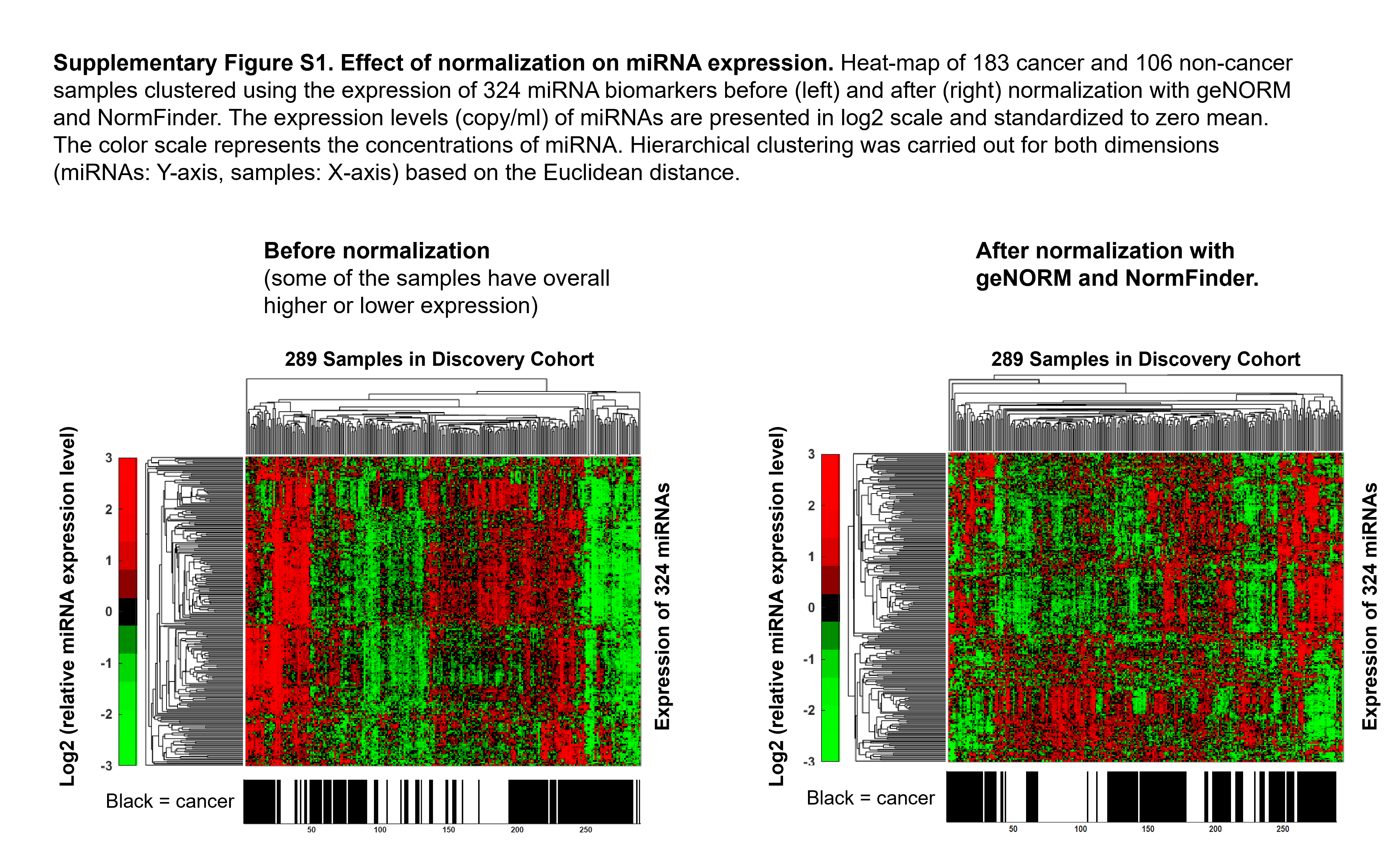

Supplement: Supplementary file 1 — Supplementary Figure S1 [file 41416_2021_1593_MOESM1_ESM.tif]
