# Supplementary material for: Development and validation of a circulating microRNA panel for the early detection of breast cancer
Source: Br J Cancer. 2022 Jan 10;126(3):472–81. doi: 10.1038/s41416-021-01593-6 (PMC8810862; doi:10.1038/s41416-021-01593-6)
Supplement: Supplementary file 2 — Supplementary Table S1 [file 41416_2021_1593_MOESM2_ESM.docx]

**Table S1. Additional information for cancer patient cohorts used in study**

| **Cohort:** | **Discovery** | **Validation 1** | **Validation 2** |
| --- | --- | --- | --- |
| **Cancer Samples:** | 183 | 177 | 180 |
| **Estrogen Receptor (ER) Status** |  |  |  |
| ER Positive | 90 (49.2%) | 78 (44.1%) | 95 (52.8%) |
| ER Negative | 93 (50.8%) | 37 (20.9%) | 18 (10.0%) |
| Unknown | 0 (0%) | 62 (35.0%) | 67 (37.2%) |
| **Progesterone Receptor (PR) Status** |  |  |  |
| PR Positive | 90 (49.2%) | 67 (37.9%) | 77 (42.8%) |
| PR Negative | 93 (50.8%) | 48 (27.1%) | 36 (20.0%) |
| Unknown | 0 (0%) | 62 (35.0%) | 67 (37.2%) |
| **HER2 Status** |  |  |  |
| HER2 Positive | 46 (25.1%) | 29 (16.4%) | 18 (10.0%) |
| HER2 Negative | 137 (74.9%) | 53 (29.9%) | 62 (34.4%) |
| HER2 Equivocal | 0 (0%) | 13 (7.3%) | 12 (6.7%) |
| Unknown | 0 (0%) | 62 (35.0%) | 67 (37.2%) |
| **Subtype** |  |  |  |
| Luminal A | 90 (49.2%) | 47 (26.6%) | 57 (31.7%) |
| Luminal B | 0 (0%) | 10 (5.6%) | 10 (5.6%) |
| Triple Negative (TNBC) | 47 (25.7%) | 6 (3.4%) | 5 (2.8%) |
| HER2-enriched | 46 (25.1%) | 19 (10.7%) | 8 (4.4%) |
| Unknown | 0 (0%) | 95 (53.7%) | 100 (55.6%) |
