# Supplementary material for: Development and validation of a circulating microRNA panel for the early detection of breast cancer
Source: Br J Cancer. 2022 Jan 10;126(3):472–81. doi: 10.1038/s41416-021-01593-6 (PMC8810862; doi:10.1038/s41416-021-01593-6)
Supplement: Supplementary file 3 — Supplementary Table S2 [file 41416_2021_1593_MOESM3_ESM.docx]

| **Supplementary Table S2 - 324 miRNAs profiled in Discovery Cohort.** |
| --- |
| hsa-miR-7a-3p |
| hsa-miR-7a-5p |
| hsa-miR-7b-3p |
| hsa-miR-7b-5p |
| hsa-miR-7d-3p |
| hsa-miR-7d-5p |
| hsa-miR-7e-3p |
| hsa-miR-7f-1-3p |
| hsa-miR-7f-5p |
| hsa-miR-7g-3p |
| hsa-miR-7g-5p |
| hsa-miR-7i-5p |
| hsa-miR-1-3p |
| hsa-miR-101-3p |
| hsa-miR-101-5p |
| hsa-miR-103a-3p |
| hsa-miR-106a-5p |
| hsa-miR-106b-3p |
| hsa-miR-106b-5p |
| hsa-miR-107 |
| hsa-miR-10a-3p |
| hsa-miR-10a-5p |
| hsa-miR-10b-5p |
| hsa-miR-1207-5p |
| hsa-miR-1208 |
| hsa-miR-122-5p |
| hsa-miR-1226-3p |
| hsa-miR-124-5p |
| hsa-miR-125a-3p |
| hsa-miR-125a-5p |
| hsa-miR-125b-5p |
| hsa-miR-126-3p |
| hsa-miR-126-5p |
| hsa-miR-127-3p |
| hsa-miR-1271-5p |
| hsa-miR-1275 |
| hsa-miR-128-3p |
| hsa-miR-1280 |
| hsa-miR-1284 |
| hsa-miR-1285-3p |
| hsa-miR-1290 |
| hsa-miR-1291 |
| hsa-miR-1299 |
| hsa-miR-130a-3p |
| hsa-miR-130b-3p |
| hsa-miR-130b-5p |
| hsa-miR-132-3p |
| hsa-miR-133a-3p |
| hsa-miR-133b |
| hsa-miR-134-5p |
| hsa-miR-135a-5p |
| hsa-miR-135b-5p |
| hsa-miR-136-3p |
| hsa-miR-136-5p |
| hsa-miR-139-5p |
| hsa-miR-140-3p |
| hsa-miR-140-5p |
| hsa-miR-141-3p |
| hsa-miR-142-5p |
| hsa-miR-143-3p |
| hsa-miR-144-3p |
| hsa-miR-144-5p |
| hsa-miR-145-5p |
| hsa-miR-146a-5p |
| hsa-miR-146b-5p |
| hsa-miR-148a-3p |
| hsa-miR-148a-5p |
| hsa-miR-148b-3p |
| hsa-miR-148b-5p |
| hsa-miR-150-3p |
| hsa-miR-150-5p |
| hsa-miR-151a-3p |
| hsa-miR-151a-5p |
| hsa-miR-152-3p |
| hsa-miR-154-5p |
| hsa-miR-15a-3p |
| hsa-miR-15a-5p |
| hsa-miR-15b-3p |
| hsa-miR-15b-5p |
| hsa-miR-16-2-3p |
| hsa-miR-16-5p |
| hsa-miR-17-3p |
| hsa-miR-17-5p |
| hsa-miR-181a-2-3p |
| hsa-miR-181a-5p |
| hsa-miR-181b-5p |
| hsa-miR-181d-5p |
| hsa-miR-182-3p |
| hsa-miR-1825 |
| hsa-miR-183-5p |
| hsa-miR-185-5p |
| hsa-miR-186-5p |
| hsa-miR-18a-3p |
| hsa-miR-18a-5p |
| hsa-miR-18b-5p |
| hsa-miR-191-5p |
| hsa-miR-192-5p |
| hsa-miR-193a-5p |
| hsa-miR-193b-3p |
| hsa-miR-194-5p |
| hsa-miR-195-5p |
| hsa-miR-196a-5p |
| hsa-miR-196b-3p |
| hsa-miR-196b-5p |
| hsa-miR-197-3p |
| hsa-miR-1973 |
| hsa-miR-199a-3p |
| hsa-miR-199a-5p |
| hsa-miR-199b-3p |
| hsa-miR-199b-5p |
| hsa-miR-19a-3p |
| hsa-miR-19b-3p |
| hsa-miR-200a-3p |
| hsa-miR-200b-3p |
| hsa-miR-200c-3p |
| hsa-miR-200c-5p |
| hsa-miR-204-5p |
| hsa-miR-205-5p |
| hsa-miR-206 |
| hsa-miR-20a-5p |
| hsa-miR-20b-5p |
| hsa-miR-21-3p |
| hsa-miR-21-5p |
| hsa-miR-214-3p |
| hsa-miR-215-5p |
| hsa-miR-216a-5p |
| hsa-miR-218-5p |
| hsa-miR-219a-5p |
| hsa-miR-22-3p |
| hsa-miR-221-3p |
| hsa-miR-221-5p |
| hsa-miR-222-3p |
| hsa-miR-223-3p |
| hsa-miR-223-5p |
| hsa-miR-224-5p |
| hsa-miR-2355-3p |
| hsa-miR-2355-5p |
| hsa-miR-23a-3p |
| hsa-miR-23a-5p |
| hsa-miR-23b-3p |
| hsa-miR-23c |
| hsa-miR-24-3p |
| hsa-miR-25-3p |
| hsa-miR-25-5p |
| hsa-miR-26a-5p |
| hsa-miR-26b-3p |
| hsa-miR-26b-5p |
| hsa-miR-27a-3p |
| hsa-miR-27a-5p |
| hsa-miR-27b-3p |
| hsa-miR-28-3p |
| hsa-miR-28-5p |
| hsa-miR-299-3p |
| hsa-miR-29a-3p |
| hsa-miR-29a-5p |
| hsa-miR-29b-2-5p |
| hsa-miR-29b-3p |
| hsa-miR-29c-3p |
| hsa-miR-29c-5p |
| hsa-miR-301a-3p |
| hsa-miR-301b-3p |
| hsa-miR-30a-5p |
| hsa-miR-30b-5p |
| hsa-miR-30c-5p |
| hsa-miR-30d-3p |
| hsa-miR-30d-5p |
| hsa-miR-30e-3p |
| hsa-miR-30e-5p |
| hsa-miR-31-3p |
| hsa-miR-3117-3p |
| hsa-miR-32-5p |
| hsa-miR-320a |
| hsa-miR-320b |
| hsa-miR-320c |
| hsa-miR-320d |
| hsa-miR-320e |
| hsa-miR-324-3p |
| hsa-miR-324-5p |
| hsa-miR-326 |
| hsa-miR-328-3p |
| hsa-miR-330-3p |
| hsa-miR-331-5p |
| hsa-miR-335-3p |
| hsa-miR-335-5p |
| hsa-miR-337-3p |
| hsa-miR-337-5p |
| hsa-miR-338-3p |
| hsa-miR-338-5p |
| hsa-miR-339-3p |
| hsa-miR-339-5p |
| hsa-miR-33a-5p |
| hsa-miR-340-5p |
| hsa-miR-342-5p |
| hsa-miR-345-5p |
| hsa-miR-346 |
| hsa-miR-34a-3p |
| hsa-miR-34a-5p |
| hsa-miR-34b-3p |
| hsa-miR-34b-5p |
| hsa-miR-361-5p |
| hsa-miR-362-3p |
| hsa-miR-362-5p |
| hsa-miR-363-3p |
| hsa-miR-365a-3p |
| hsa-miR-365b-5p |
| hsa-miR-369-3p |
| hsa-miR-369-5p |
| hsa-miR-370-3p |
| hsa-miR-374a-3p |
| hsa-miR-374a-5p |
| hsa-miR-374b-3p |
| hsa-miR-374b-5p |
| hsa-miR-374c-5p |
| hsa-miR-375 |
| hsa-miR-376a-5p |
| hsa-miR-376b-3p |
| hsa-miR-377-3p |
| hsa-miR-378a-3p |
| hsa-miR-378a-5p |
| hsa-miR-379-5p |
| hsa-miR-381-3p |
| hsa-miR-382-5p |
| hsa-miR-409-3p |
| hsa-miR-411-3p |
| hsa-miR-411-5p |
| hsa-miR-421 |
| hsa-miR-423-3p |
| hsa-miR-423-5p |
| hsa-miR-424-5p |
| hsa-miR-425-3p |
| hsa-miR-425-5p |
| hsa-miR-4257 |
| hsa-miR-429 |
| hsa-miR-4306 |
| hsa-miR-431-3p |
| hsa-miR-431-5p |
| hsa-miR-432-5p |
| hsa-miR-450a-5p |
| hsa-miR-451a |
| hsa-miR-452-5p |
| hsa-miR-454-3p |
| hsa-miR-454-5p |
| hsa-miR-455-5p |
| hsa-miR-4732-3p |
| hsa-miR-4732-5p |
| hsa-miR-483-3p |
| hsa-miR-483-5p |
| hsa-miR-484 |
| hsa-miR-485-3p |
| hsa-miR-485-5p |
| hsa-miR-486-5p |
| hsa-miR-487b-3p |
| hsa-miR-491-5p |
| hsa-miR-493-3p |
| hsa-miR-493-5p |
| hsa-miR-495-3p |
| hsa-miR-497-5p |
| hsa-miR-499a-5p |
| hsa-miR-500a-3p |
| hsa-miR-500a-5p |
| hsa-miR-501-3p |
| hsa-miR-501-5p |
| hsa-miR-502-3p |
| hsa-miR-503-5p |
| hsa-miR-505-3p |
| hsa-miR-513a-5p |
| hsa-miR-518b |
| hsa-miR-519a-5p |
| hsa-miR-520d-5p |
| hsa-miR-523-5p |
| hsa-miR-532-3p |
| hsa-miR-532-5p |
| hsa-miR-539-5p |
| hsa-miR-550a-5p |
| hsa-miR-551b-3p |
| hsa-miR-551b-5p |
| hsa-miR-571 |
| hsa-miR-573 |
| hsa-miR-576-5p |
| hsa-miR-579-3p |
| hsa-miR-584-5p |
| hsa-miR-589-5p |
| hsa-miR-590-5p |
| hsa-miR-596 |
| hsa-miR-598-3p |
| hsa-miR-609 |
| hsa-miR-610 |
| hsa-miR-616-3p |
| hsa-miR-616-5p |
| hsa-miR-618 |
| hsa-miR-620 |
| hsa-miR-621 |
| hsa-miR-625-5p |
| hsa-miR-627-5p |
| hsa-miR-628-5p |
| hsa-miR-629-3p |
| hsa-miR-629-5p |
| hsa-miR-646 |
| hsa-miR-650 |
| hsa-miR-651-5p |
| hsa-miR-652-3p |
| hsa-miR-654-3p |
| hsa-miR-654-5p |
| hsa-miR-660-5p |
| hsa-miR-663a |
| hsa-miR-668-3p |
| hsa-miR-671-3p |
| hsa-miR-7-5p |
| hsa-miR-720 |
| hsa-miR-769-5p |
| hsa-miR-874-3p |
| hsa-miR-885-5p |
| hsa-miR-9-3p |
| hsa-miR-9-5p |
| hsa-miR-92a-3p |
| hsa-miR-92b-3p |
| hsa-miR-93-3p |
| hsa-miR-93-5p |
| hsa-miR-95-3p |
| hsa-miR-96-5p |
| hsa-miR-98-5p |
| hsa-miR-99a-5p |
| hsa-miR-99b-3p |
| hsa-miR-99b-5p |
