# Supplementary material for: Development and validation of a circulating microRNA panel for the early detection of breast cancer
Source: Br J Cancer. 2022 Jan 10;126(3):472–81. doi: 10.1038/s41416-021-01593-6 (PMC8810862; doi:10.1038/s41416-021-01593-6)
Supplement: Supplementary file 4 — Supplementary Table S3 [file 41416_2021_1593_MOESM4_ESM.docx]

| **Supplementary Table S3 - Relative expression levels and individual performance of the miRNAs included in the optimal eight-miRNA panel** | | | | | | | | | | |
| --- | --- | --- | --- | --- | --- | --- | --- | --- | --- | --- |
|  | | | | | | | | | |  |
|  | **miRNA biomarker:** | **miR-377-3p** | **miR-374c-5p** | **miR-324-5p** | **miR-24-3p** | **miR-133a-3p** | **miR-125b-5p** | **miR-497-5p** | **miR-19b-3p** |  |
| ***p*-value (cancer vs non-cancer)** | **Discovery Cohort** | 1.01E-09 | 2.39E-13 | 1.97E-17 | 4.70E-25 | 8.32E-08 | 1.48E-14 | 1.74E-15 | 2.17E-12 |  |
|  | **Validation 1 Cohort** | 8.09E-03 | 1.29E-11 | 9.55E-12 | 1.42E-13 | 6.42E-04 | 3.91E-04 | 1.87E-03 | 5.85E-05 |  |
|  | **Validation 2 Cohort** | 3.45E-02 | 8.97E-13 | 2.88E-14 | 1.49E-20 | 5.14E-02 | 2.59E-04 | 2.08E-02 | 1.22E-05 |  |
| **log2(Fold-Change) (cancer vs non-cancer)** | **Discovery Cohort** | 0.5684 | -1.1143 | -0.7877 | 0.9359 | 1.3679 | 1.0654 | 0.7419 | 0.6982 |  |
|  | **Validation 1 Cohort** | 0.1886 | -0.9278 | -0.6003 | 0.5066 | 0.5592 | 0.4145 | 0.1684 | 0.246 |  |
|  | **Validation 2 Cohort** | 0.1466 | -0.9892 | -0.6709 | 0.6196 | 0.3056 | 0.4079 | 0.1319 | 0.2584 |  |
| **AUC** | **Discovery Cohort** | 0.7082 | 0.7658 | 0.8186 | 0.8478 | 0.6513 | 0.767 | 0.7972 | 0.7465 |  |
|  | **Validation 1 Cohort** | 0.5927 | 0.7009 | 0.6908 | 0.7175 | 0.5665 | 0.6098 | 0.5874 | 0.6195 |  |
|  | **Validation 2 Cohort** | 0.5558 | 0.7047 | 0.7262 | 0.7591 | 0.5224 | 0.6245 | 0.5809 | 0.6303 |  |
